# What Does “High Risk” Mean in Surgical Patients?

**DOI:** 10.1002/deo2.70323

**Published:** 2026-04-06

**Authors:** Ece Salihoglu, Ziya Salihoglu

**Affiliations:** ^1^ Cardiovascular Surgery Department İstanbul Nisantasi University Istanbul Turkey; ^2^ Anesthesiology and Reanimation Department Cerrahpasa Medical Faculty İstanbul University‐Cerrahpasa Istanbul Turkey

To the Editor:

We read the article by Maruta et al. titled “Endoscopic management of acute cholecystitis in high‐risk surgical patients” [1].

This review summarizes the characteristics of each drainage method and compares the clinical outcomes of the three procedures for acute cholecystitis in high‐risk surgical patients.

The authors defined high‐risk patients only as “advanced age, deteriorated performance status, or underlying diseases, conservative treatment”. They did not discuss the criteria to define high‐risk patients, the inclusion and exclusion criteria, and major and minor morbidities in surgery, anesthesia, and the perioperative period.

Perioperative risk classification of patients undergoing anesthesia is of essential interest and has been the focus of much research and categorization. Commonly, these investigations were performed by analyzing clinical experiences and large patient series. The ideal categorization system, based on medical doctors’ clinical experiences and in cooperation with other disciplines, has not been developed yet.

There are increasing numbers of studies on laparoscopic surgery, but there are few studies on the case of high‐risk patients. In some studies, a relationship between pre‐operative and postoperative patient information was examined, and an approach was proposed for the selection of surgery type. As stated formerly, although there are studies to determine the risks in Laparoscopic operations. There is no general model to determine the high‐risk patient before the surgery [2]. This article does not adequately evaluate high‐risk cases [1]. According to the Tokyo 2018 Guidelines, high‐risk patients for emergency laparoscopic cholecystectomy in acute cholecystitis have been defined [3].

The Charlson Comorbidity Index is a widely validated, weighted scoring system used to predict 1‐ or 10‐year mortality. The American Society of Anesthesiologists (ASA) classification is the most commonly used classification for assessing preoperative health status. The ASA classification is only an indicator of physical condition; it is not a risk classification. This subjective classification is based on the patient's self‐report [3].

“In considering drainage strategies for acute cholecystitis, a more detailed definition and discussion of ‘high‐risk’ patients remains an important issue.”

## Author Contributions

Conceptualization: Salihoğlu Z. Writing – review & editing: Salihoğlu E.

## Funding

The authors have nothing to report.

## Conflicts of Interest

The authors declare no conflicts of interest.
